# Common extensor origin release in recalcitrant lateral epicondylitis - role justified?

**DOI:** 10.1186/1749-799X-5-31

**Published:** 2010-05-10

**Authors:** Faizal Rayan, Vittal SR Rao, Sanjay Purushothamdas, Cibu Mukundan, Syed O Shafqat

**Affiliations:** 1 Department of Trauma & Orthopaedics, University College Hospital, London UK; 2 Department of Surgery, Scunthorpe General Hospital, Scunthorpe, UK; 3 Nuffield Orthopaedic Spine centre, Oxford, UK; 4 Department of Trauma & Orthopaedics, Scarborousgh Hospital, Scarborough, UK; 5 Department of Trauma & Orthopaedics, Scunthorpe General Hospital, Scunthorpe, UK

## Abstract

The aim of our study was to analyse the efficacy of operative management in recalcitrant lateral epicondylitis of elbow. Forty patients included in this study were referred by general practitioners with a diagnosis of tennis elbow to the orthopaedic department at a district general hospital over a five year period. All had two or more steroid injections at the tender spot, without permanent relief of pain. All subsequently underwent simple fasciotomy of the extensor origin. Of forty patients thirty five had improvement in pain and function, two had persistent symptoms and three did not perceive any improvement. Twenty five had excellent, ten had well, two had fair and three had poor outcomes (recurrent problem; pain at rest and night). Two patients underwent revision surgery. Majority of the patients had improvement in pain and function following operative treatment. In this study, an extensor fasciotomy was demonstrated to be an effective treatment for refractory chronic lateral epicondylitis; however, further studies are warranted.

## Introduction

Lateral epicondylitis is characterised by localised pain over the origin of extensor muscles of the finger and wrist at the lateral epicondyle. The cornerstone of the diagnosis are detailed history regarding aggravating and relieving factors and the provocative tests like grasping in elbow extension, resisted wrist and long finger extension and resisted forearm supination[1]. There is often a decrease in the grip strength[1]. Differential diagnosis includes radial tunnel syndrome, radio humeral arthritis, osteochondritis of capitellum, posterolateral instability of the elbow and injury to lateral ante brachial cutaneous nerve [1-3]. An AP, lateral and radiocapitellar view are used as primary imaging modality in order to rule out intraarticular disease or a musculoskeletal tumor. The other imaging techniques like magnetic resonance imaging, electromyography and nerve conduction studies may be complementary[1].

Most of the current non-operative modalities utilized in the treatment on lateral epicondylitis are not evidence based[1]. Most of the studies do not differentiate between clinical and statistical significance, and they were unable to depict any beneficial effect of their treatment over natural history of the condition. Patients who fail to respond to conservative measures may require surgery (<10%)[2]. Various operative techniques including open, percutaneous and arthroscopic techniques have been described[2]. Percutaneous procedure has an advantage of reduced morbidity but it has an inherent possibility of inadequate resection or recurrence [4]. Also it is not possible to rule out intraarticular pathology (concurrence of 18.8%)[5]. In the literature there is only one study comparing all three techniques, even though it was done retrospectively[2].

## Materials and methods

In a 5 year period 40 patients referred by general practitioners as tennis elbow who had surgical intervention were reviewed in this retrospective study. The surgery was performed by the senior author. The inclusion criteria were 1. Clinical diagnosis (tenderness on provocative tests) 2. Patients who had failed conservative treatment (All patients had two or more cortisone injections, splints, oral anti-inflammatory agents and activity modification before the operation. They also received physiotherapy in the form of heat, ultrasound and muscle strengthening exercises. All had 2 or more cortisone injections at the tender spot with recurrence of symptoms). The exclusion criteria were 1. Patients with neck pain 2. Patients with inflammatory arthropathy 3. History of trauma. All patients included in this study had telephonic questionnaire or follow up examination. Demographics, medical history, co-existing orthopaedic problems and surgical findings were gleaned from the records. Dominant limb was involved in 34 patients with duration of symptoms for more than 12 months. All the 40 refractory cases underwent simple fasciotomy of the extensor origin. Results were rated as per Grundberg. (Table 1) This is a simple grading system where they are graded into 4 based on level of satisfaction, pain and activity level postoperatively. All patients who were satisfied and returned to work without any pain were graded as excellent. Patients who were satisfied and returned to work with pain only during heavy use were graded as good. Partially satisfied patients returned to work with activity limited by pain was graded as fair. Those who were dissatisfied had persistent pain were graded as poor. Patients were asked to verbally rate his or her level of perceived pain intensity, both pre and postoperatively on a numerical scale from 0 to 10 with zero representing no pain and 10 representing the other extreme. The hypothesis of our study was simple fasciotomy of the extensor origin is a viable option in the treatment of recalcitrant lateral epicondylitis.

**Table 1:** Patients rated as per Grundberg criteria

Rating	Pain	Patient satisfaction
Excellent = 25	No pain	Returned to activity/ Patient satisfied

Good = 10	Pain with heavy use	Returned to activity/ Patient satisfied

Fair = 2	Pain which limits activity	Returned to activity/ Patient partially satisfied

Poor = 3	Pain unchanged	Patient dissatisfied

### Surgical Procedure

Under general anaesthesia and tourniquet control a curvilinear incision centred over lateral epicondyle measuring approximately one inch is made, the deep fascia is incised distal to lateral epicondyle. Common extensor origin (extensor carpi radialis longus, brevis, extensor digitorum and the anconeus) is released and retracted. Care was taken to avoid any damage to lateral collateral ligament. Wound was closed in layers using 4-0 monocryl for the skin. Wool and crepe dressing with elbow flexed at 90 degrees was applied. Patients were encouraged to start activities within limits of pain.

## Results

Majority of the patients were in the fifth decade, the age range was 31-60 years with an average of 43.7 years and Male: Female ratio was 16:24. The minimum follow up was 12 months, the range was 12 to 54 months with an average follow up period of 24 months No patient were lost to follow up There were no post operative complications. Thirty five patients had full range of movements post operatively. The mean preoperative pain score was 8.9 and the mean post operative pain score was 1.6. Full range of painless motion was achieved. Out of 40 patients 35 had significant improvement in pain and function, two had persistent symptoms and three did not perceive any improvement. Twenty five had excellent, ten had good, two had fair and 3 had poor outcomes (recurrent problem; pain at rest and night). Two patients underwent revision surgery with satisfying results. During revision, excessive scar tissue was excised and the extensor aponeurosis was repaired. Care was taken to stay superficial during dissection to avoid any damage to lateral collateral ligament, and none of them had posterolateral rotatory instability of the elbow. Majority of the patients had significant improvement following operative treatment.

## Discussion

Lateral epicondylitis or tennis elbow is one of the most regularly encountered disorders of the elbow that can cause significant pain and dysfunction. This disorder was first described by Runge in 1873 and the term tennis elbow was coined in 1883 by Major [6,7]. Both the terms are misnomers as it occurs more commonly in non athletes and there is a contrasting evidence to suggest there is an inflammatory process[6]. Less than 5-10% of patients with lateral tennis elbow syndrome are tennis players, however as group tennis players do run a higher risk of developing this syndrome [8,9]. The incidence of tennis elbow varies from 1-3% [10]. It is seen more often in fourth decade [10]. Even if aetiology is attributed to various factors like bursitis, synovitis, ligament inflammation, periosteitis; the most common accepted etiology is microscopic tears with formation of reparative tissue on the lateral epicondyle[11]. Cyriax in 1936 had explained about 26 etiological factors of tennis elbow[2]. Still the pathology is uncertain as no published data have examined patients with acute diagnosis of tennis elbow [4,12]. The natural history of this disease is 70-80% resolution at 1 year [10,13].

The number and variety of overuse syndromes are expected to increase since our lifestyle demands physical fitness and sports participation. The principle of any successful operation is the accurate identification of the pathological process involved and its correction with a minimum disruption of normal tissues. The treatment of tennis elbow has been laden with controversy. Various surgical techniques including fasciotomy, z-lengthening of the tendon, osteotomy of the lateral epicondyle and excision of the damaged portion of ECRB(extensor carpi radialis brevis) as well open and percutaneous tenotomy have been described in the literature. Arthroscopic technique is also in vogue. Poor surgical outcome due to the operative intervention can be correlated with residual tendinopathy [14]. No conclusions were derived from the last Cochrane database review of tennis elbow; we have a dearth of level II evidence to substantiate the advantage of any one surgical treatment for tennis elbow. So far in the literature we could trace only one randomized controlled trial comparing open, percutaneous and arthroscopic techniques in the treatment of tennis elbow[15].

Grundberg and Dobson reported good or excellent results in 29 of 32 elbows and Baumguard and Schwartz achieved 32 excellent and three dissatisfied patients in 35 elbows following percutaneous release [2,16]. Nirschl and Pettrone achieved an excellent outcome in 66 of 88 elbows using an open technique[17,11]. The reported rates of good results have ranged from 54% to 99% in common extensor origin release which makes it an exceedingly reasonable procedure for the treatment of lateral epicondylitis unresponsive to conservative treatment[6].

## Conclusion

Our results demonstrated no complications and a low rate of revision surgeries in those patients treated with this technique (2/40) with a success rate on par with previous published series on other techniques for surgical treatment of lateral epicondylitis. The main drawbacks of this study are the retrospective nature, smaller number of patients, lack of comparison group and the duration of follow up. However they were treated by a single surgeon with a standard technique. We were able to obtain follow-up data on all of the original 40 elbows studied. The results suggest that a release of the common extensor tendon origin may be a reasonable approach to the treatment of lateral epicondylitis and should be evaluated further as part of a more controlled randomized investigation.

## Competing interests

The authors declare that they have no competing interests.

## Authors' contributions

FR Conception, Data collection and drafting the manuscript

VSRR Conception, Designing, data collection and analysis

SP Data analysis and drafting the manuscript.

CM Data analysis and drafting the manuscript

OS Conception, Data collection, Coordination and drafting the manuscript

All authors read and approved the final manuscript.

## References

[B1] Boyer MI Hastings H Lateral tennis elbow: "Is there any science out there?" J Shoulder Elbow Surg 1999 8 481 491 10.1016/S1058-2746(99)90081-2 10543604

[B2] Lo MY Safran MR Surgical treatment of lateral epicondylitis: a systematic review Clin Orthop Relat Res 2007 463 98 106 17632419 10.1097/BLO.0b013e3181483dc4

[B3] Pomerance J Radiographic analysis of lateral epicondylitis J Shoulder Elbow Surg 2002 11 156 157 10.1067/mpn.2002.121147 11988727

[B4] Yerger B Turner T Percutaneous extensor tenotomy for chronic tennis elbow: an office procedure Orthopedics 1985 8 1261 1263 4094961 10.3928/0147-7447-19851001-11

[B5] Owens BD Murphy KP Kuklo TR Arthroscopic release for lateral epicondylitis Arthroscopy 2001 17 582 587 11447544 10.1053/jars.2001.20098

[B6] Kaleli T Ozturk C Temiz A Tirelioglu O Surgical treatment of tennis elbow: percutaneous release of the common extensor origin Acta Orthop Belg 2004 70 131 133 15165014

[B7] Rayan GM Coray SA V-Y slide of the common extensor origin for lateral elbow tendonopathy J Hand Surg [Am] 2001 26 1138 1145 10.1053/jhsu.2001.28432 11721266

[B8] Gruchow HW Pelletier D An epidemiologic study of tennis elbow. Incidence, recurrence, and effectiveness of prevention strategies Am J Sports Med 1979 7 234 238 10.1177/036354657900700405 474862

[B9] Assendelft WJ Hay EM Adshead R Bouter LM Corticosteroid injections for lateral epicondylitis: a systematic overview Br J Gen Pract 1996 46 209 216 8703521 PMC1239602

[B10] De Smedt T de Jong A Van Leemput W Lieven D Van Glabbeek F Lateral epicondylitis in tennis: update on aetiology, biomechanics and treatment Br J Sports Med 2007 41 816 819 10.1136/bjsm.2007.036723 17616547 PMC2465303

[B11] Nirschl RP Pettrone FA Tennis elbow. The surgical treatment of lateral epicondylitis J Bone Joint Surg Am 1979 61 832 839 479229

[B12] Coonrad RW Hooper WR Tennis elbow: its course, natural history, conservative and surgical management J Bone Joint Surg Am 1973 55 1177 1182 4758032

[B13] Labelle H Guibert R Joncas J Newman N Fallaha M Rivard CH Lack of scientific evidence for the treatment of lateral epicondylitis of the elbow. An attempted meta-analysis J Bone Joint Surg Br 1992 74 646 651 1388172 10.1302/0301-620X.74B5.1388172

[B14] Cummins CA Lateral epicondylitis: in vivo assessment of arthroscopic debridement and correlation with patient outcomes Am J Sports Med 2006 34 1486 1491 10.1177/0363546506288016 16685085

[B15] Dunkow PD Jatti M Muddu BN A comparison of open and percutaneous techniques in the surgical treatment of tennis elbow J Bone Joint Surg Br 2004 86 701 704 10.1302/0301-620X.86B5.14469 15274267

[B16] Grundberg AB Dobson JF Percutaneous release of the common extensor origin for tennis elbow Clin Orthop Relat Res 2000 137 140 10.1097/00003086-200007000-00019 10906868

[B17] Nirschl RP Lateral extensor release for tennis elbow J Bone Joint Surg Am 1994 76 951 8200903 10.2106/00004623-199406000-00021

